# Prevalence and Correlates of Cardiovascular Autonomic Neuropathy Among Patients with Diabetes in Uganda: A Hospital-Based Cross-sectional Study

**DOI:** 10.5334/gh.765

**Published:** 2020-03-02

**Authors:** Richard Migisha, David Collins Agaba, Godfrey Katamba, Teddy Kwaga, Raymond Tumwesigye, Silvia Lopez Miranda, Anthony Muyingo, Mark J. Siedner

**Affiliations:** 1 Department of Physiology, Mbarara University of Science and Technology, Mbarara, UG; 2 Department of Physiology, St. Augustine International University, Kampala, UG; 3 Department of Ophthalmology, Mbarara University of Science and Technology, Mbarara, UG; 4 Department of Nursing, Mbarara University of Science and Technology, Mbarara, UG; 5 Department of Internal Medicine, Mbarara University of Science and Technology, Mbarara, UG; 6 Department of Medicine, Massachusetts General Hospital, Boston, US

**Keywords:** Cardiovascular autonomic neuropathy, Prevalence, Diabetes, Uganda

## Abstract

**Background:**

Cardiovascular autonomic neuropathy (CAN) is a common complication in individuals with diabetes mellitus (DM) but often overlooked in clinical practice. The burden and correlates of CAN have not been extensively studied in low-income countries, particularly in sub-Saharan Africa.

**Objectives:**

To determine the prevalence and correlates of CAN among adults in ambulatory diabetes care in southwestern Uganda.

**Method:**

We conducted a cross-sectional study among adults with diabetes from November 2018 to April 2019. CAN was assessed using the five autonomic function tests: deep breathing, Valsalva maneuver, postural index on standing, change in blood pressure during standing and diastolic blood pressure response to isometric exercise. We estimated the prevalence of CAN and fit regression models to identify its demographic and clinical correlates.

**Results:**

We enrolled 299 individuals. The mean age was 50.1 years (SD ± 9.8), mean HbA1c was 9.7 (SD ± 2.6) and 69.6% were female. CAN was detected in 156/299 (52.2%) of the participants on the basis of one or more abnormal cardiovascular autonomic reflex tests. Out of 299 participants, 88 (29.4%) were classified as early CAN while 61/299 (20.4%) and 7/299 (2.3%) were classified as definite and severe (advanced) CAN respectively. In multivariable regression models, age over 50 years (aOR 3.48, 95%CI 1.35 –8.99, p = 0.010), duration of diabetes over 10 years (aOR 4.09, 95%CI 1.78 –9.38, p = 0.001), and presence of diabetic retinopathy (aOR 2.25, 95%CI 1.16 –4.34, p = 0.016) were correlated with CAN.

**Conclusions:**

Our findings reveal a high prevalence of CAN among individuals in routine outpatient care for diabetes mellitus in Uganda. Older age, longer duration of diabetes and coexistence of retinopathy are associated with CAN. Future work should explore the clinical significance and long term outcomes associated with CAN in this region.

## Background

The global prevalence of diabetes is 8.5%, with an estimated 422 million people living with the disease [1]. As of 2014, the prevalence of diabetes was higher in low-income (7.4%) than in high-income (7.0%) countries [2]. Moreover, it is estimated that the disease burden will increase by approximately 70% in low income countries by 2030 [3,4]. Sub-Saharan Africa, in particular, is believed to have the highest proportion of undiagnosed diabetes with more than two-thirds of people with diabetes unaware of their status [5,6].

Cardiovascular autonomic neuropathy (CAN) is defined as an impairment of cardiovascular autonomic control in people with diabetes with other causes excluded [7,8]. Cardiovascular reflex tests that are based on provocative physiological maneuvers of the autonomic nervous system (Valsalva maneuver, supine to standing, deep breathing and sustained hand grip) are commonly used in the detection of CAN [9]. There is no particular algorithm for the diagnosis of CAN, though it is recommended that more than one test be carried out to increase the reliability and sensitivity for proper diagnosis of the condition [10]. The occurrence of CAN, on the basis of at two or more tests [7,11,12] ranges from approximately 20% to 65%, with older age and longer duration of disease [13,14]. CAN is associated with an increased risk of cardiac arrhythmias, silent myocardial ischemia and sudden death [10,15]. Hence, it may limit exercise capacity and is associated with an increased risk of adverse cardiovascular events during exercise [16]. Moreover CAN is associated with increased risk of mortality [17]. Previous longitudinal studies among diabetic individuals with CAN have reported five-year mortality rates of up to 50%, often due to sudden cardiac death [18]. Despite these complications, CAN remains largely undiagnosed among individuals with diabetes. Nonetheless, early detection and treatment of CAN may prevent cardiovascular complications in diabetic patients [19], since there is evidence to suggest that cardiovascular denervation can be reversed early in the course of the condition [9,20].

Despite the high prevalence of diabetes in low-resource settings, most studies on prevalence and correlates of CAN have been done in high-resource settings. There are particularly few data on the condition in Sub-Saharan Africa. In this clinic-based survey, we aimed to determine the prevalence and correlates of CAN among individuals with diabetes in ambulatory diabetes care in southwestern Uganda.

## Methods

### Study population

This cross-sectional study was conducted at the diabetes and endocrinology outpatient clinic of Mbarara Regional Referral Hospital (MRRH), Western Uganda. MRRH is a government-operated referral centre and the teaching hospital of the Medical School of Mbarara University of Science and Technology (MUST), serving a population of over four million people in its catchment area. The diabetes and endocrinology ambulatory care clinic is operated once per week and attends to an average of 100 patients per week. A total of about 1,500 diabetic patients are registered at the clinic.

Study participants were individuals with diabetes attending MRRH diabetes and endocrinology ambulatory clinic. We included participants aged 18 to 65 years with a clinical diagnosis of diabetes. We defined diabetes in those with a fasting blood glucose concentration greater than 7.0 mmol/l, or in those actively taking medications for diabetes [21,22,23]. We excluded individuals with a self-reported history of cardiac, respiratory, renal, hepatic, cerebrovascular, thyroid or other endocrine abnormalities. We also excluded individuals with previous ECG abnormalities, an acute illness in the last 48 hours, and those who had consumed beverages containing caffeine or alcohol within the past 12 hours. Finally, we excluded individuals actively taking calcium channel blockers or beta-blockers, which can interfere with the CAN screening battery [24].

### Study Procedures and Definitions

We administered a standardized questionnaire to capture data on socio-demographic and clinical characteristics, including their data on smoking, alcohol use and diabetes treatment history. The diabetes treatment history was self-reported and verified by clinic records for those participants who had a chat available for review. Height, weight, hip circumference, and waist circumference were collected. We computed body mass index (BMI) by dividing participants’ weight (in kilograms) by the height in metres squared (m^2^), and categorized BMI as normal, underweight, overweight and obese [25]. Abdominal obesity was defined as a waist circumference ≥102 cm in males and ≥88 cm in females [25]. Blood pressure was measured using an upper arm automated sphygmomanometer (Omron HEM 705 LP, Omron Healthcare, Inc., Bannockburn, IL, USA) in a seated position. Participants were considered to have arterial hypertension if their blood pressure values were ≥140/90 mmHg and/or taking medications for hypertension [26]. Intensity of physical activity was expressed in metabolic equivalent task (MET) as derived from the questionnaire using the WHO GPAQ analysis tool [27]. The presence of diabetic retinopathy was detected with direct ophthalmoscopy after pupillary dilation by an ophthalmologist. Diabetic retinopathy was classified or graded as non-proliferative (NPDR) and proliferative diabetic retinopathy (PDR). We assessed for the presence of the following symptoms of distal peripheral neuropathy over the preceding 6 months: numbness or ‘dead feeling’ in the feet; a tingling sensation in the feet; deep, painful or burning aches in the lower limbs; and unusual failure in walking uphill or climbing staircases. The existence of one or more of the symptoms was classified as abnormal. Hemoglobin A1c (HbA1c) was measured according to the standard operating procedure of the International Federation of Clinical Chemistry and Laboratory Medicine (IFCC) [28] using an automated high performance liquid chromatography analyzer (Cobas Integra 400, Roche diagnostics, Basel, Switzerland) at the Lancet Laboratories.

The evaluation of CAN was done according to standardized protocols described previously [12]. Briefly, we considered five measures to diagnosis CAN, including: 1) deep breathing; 2) Valsalva maneuver; 3) postural index on standing; 4) change in blood pressure during standing and 5) diastolic blood pressure response to isometric exercise. A resting 12-Lead electrocardiogram (ECG) was taken for all the participants using a portable ECG machine (Edan Instruments, Inc., Hessen, Germany).

#### Deep breathing

While lying down, participants were asked to perform a deep and slow inspiration up to the maximum total lung capacity for five seconds, which was followed by a forced expiration down to the residual volume for five seconds. The time to alternate the respiratory cycle was signaled directly to the patient by the attending research assistant. We calculated the maximum and minimum R-R interval during the respiratory cycle, and converted these results to beats per minute. We defined R-R interval as the duration between two successive R waves. We considered a variation of less than 15 beats per minute between the two measures as abnormal.

#### Valsalva maneuver

We asked participants to exhale into a mouthpiece with their nose closed and to perform a continuous expiratory effort. This was equivalent to an intraoral pressure of 40 mmHg, for 15 seconds. We then asked participants to release the strain, and to breathe regularly until the end of the test. We calculated the ratio of the longest RR interval to the shortest RR interval following the pressure release. We considered a ratio less than 1.2 as abnormal. The test was performed three times, and we used the mean value in our analyses. Participants with evidence of proliferative retinopathy were excluded from the valsalva maneuver, because it is associated with a minor risk of intraocular hemorrhage or lens dislocation [7].

#### Measurement of postural index on standing

We again asked participants to lie supine quietly. After about 5 minutes with continuous monitoring, the participant was made to stand. We calculated the postural index (PI) by measuring the heart rate response to the change of position from recumbent to standing over 180 seconds after getting up. We calculated this as the ‘30–15 ratio,’ defined as the longest RR interval of beats 20–40 divided by the shortest RR interval of beats 5–25, starting from the first beat during the process of standing up. The postural index of <1.00 was considered abnormal.

#### Change in blood pressure during standing

We measured blood pressure with participants in recumbent position, every minute for three minutes, and then while standing, every minute for five minutes. We considered a drop of ≥20 mmHg in systolic blood pressure or ≥10 mmHg in diastolic blood pressure as abnormal.

#### Diastolic blood pressure response to isometric exercise

We asked participants to squeeze a hydraulic hand dynamometer (Lafayette Instrument Company, Lafayette, IN, USA) at 1/3 of the maximum effort for about 5 minutes. We considered an abnormal diastolic blood pressure in response to isometric exercise as an increase in diastolic blood pressure <15 mmHg.

### Measurement of QT interval

We measured the QT interval from the beginning of the QRS complex to the end of the down-slope of the T wave (crossing of the isoelectric line); when a U wave was present, the QT interval was measured until the bottom of the angle between the T and U waves [29]. We used Bazette’s formula to calculate a heart rate–corrected QT (QTc) [30]. QTc was the mean of QTc from five consecutive cycles in lead V5. We consider a maximum value of the QTc interval >440 ms to be elevated [31]. For quality control, two independent observers (DCA and GK) measured intervals. Both were blinded to participants’ data, and an average measure taken. The Pearson’s correlation coefficients for the agreement between the two observers were 0.92 and 0.87 for QTc and RR Intervals respectively. We performed ECG on the same day as glycaemic testing.

### Sample size

A sample size of 296 participants was required to enable a 5% precision with a 95% confidence interval around an estimated CAN prevalence of 20% [32]. We entered data into EpiData3. (EpiData, Odense, Denmark), and used Stata for all analyses (StataCorp, College Station, Texas, USA).

### Statistical Analyses

Our outcome of interest was CAN, defined by the results of each of the above five classification components. We categorized CAN based on severity, as described previously (Table 1 [33]). We summed the points from each test to arrive at an overall CAN score, categorized as absent (0 points), early (0.5–1.5 points), definite (2–3 points), and severe (≥3.5 points) [12,34].

**Table 1:** Algorithm for classification of CAN (CAN score).

Tests	Point 0	Point 0.5	Point 1

Resting heart rate	<100 beats/min	100–110 beats/min	>110 beats/min
Postural hypotension (fall in systolic blood pressure)	<20 mmHg	20–30 mmHg	>30 mmHg
Valsalva ratio	>1.2	1.2–1.10	<1.10
Heart rate variability on deep breathing	>15 beats/min	15–10 beats/min	<10 beats/min
Increase in diastolic blood pressure during sustained handgrip	>15 mmHg	15–10 mmHg	<10 mmHg

CAN: *Cardiovascular autonomic neuropathy.*

We first described the participant characteristics. Next we estimated the prevalence of CAN as the proportion of patients meeting the definition of CAN. We then assessed for differences in demographic and clinical characteristics comparing those with and without CAN, using chi-square testing to compare categorical variables, student t-tests for continuous variables, and Wilcoxon rank-sum test for non-normally distributed continuous variables. Finally, we fit logistic regression models to identify correlates of CAN. Covariates with a *P-*value ≤ 0.2 in univariable models were included in multivariable models through backward stepwise elimination method. QTc interval was deliberately not included in the final model because it predicted the outcome perfectly.

## Results

**Figure 1:**
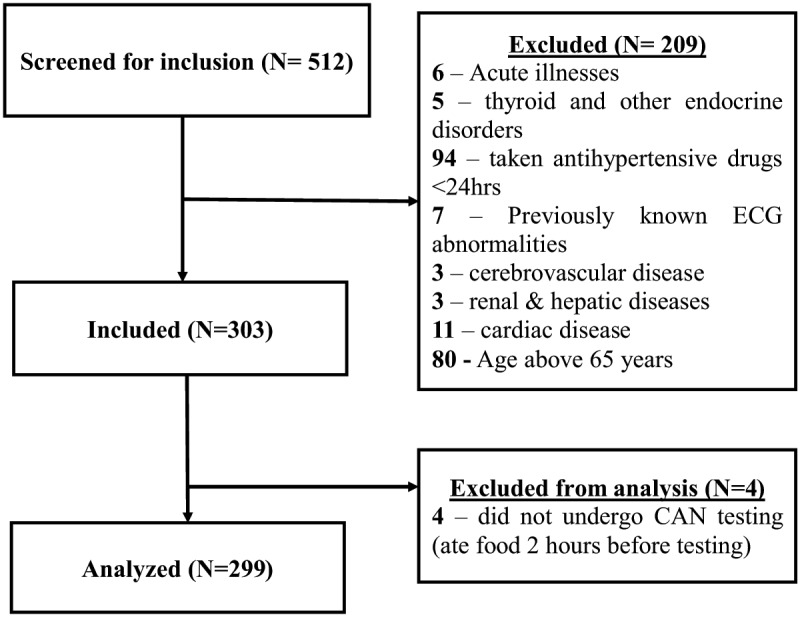
Study profile.

Out of the 512 diabetic patients screened for inclusion into the study between November 2018 and April 2019, we present results for 299 participants. The reasons for exclusion are described in Figure 1.

### Demographic and clinical characteristics

Social and demographic characteristics of study participants with and without CAN are presented in Table 2. Most participants were female (69.6%) and peasants by occupation (58.6%). More than half (69.9%) of the participants had never taken alcohol. The mean age of the participants was 50.1 (SD ± 9.8) years. Seventy participants (23.4%) had ever smoked. The clinical characteristics of the participants with and without CAN are presented in Table 2. The mean duration of diabetes was four years (IQR 1, 8). The mean fasting blood glucose and HbA1c were 11.2 mmol/l (SD ± 4.8) and 9.7% (SD ± 2.6), respectively. Out of the 299 participants, 68 (22.7%) had diabetic retinopathy.

**Table 2:** Baseline demographic and clinical characteristics by CAN status.

Characteristic	Total cohort (N = 299)	Positive for CAN (N = 156)	No evidence of CAN (N = 143)	P value

**Age, in years, mean (SD)**	50.1 (±9.8)	52.8 (±8.7)	47.2 (±10.2)	<0.001
**Female sex (%)**	208 (69.6)	122 (78.2)	86 (60.1)	0.001
**Ever attended school, yes (%)**	233 (77.9)	115 (73.7)	118 (82.5)	0.067
**Occupation**				
Peasant (%)	175 (58.5)	103 (66.0)	72 (50.4)	0.006
Student (%)	6 (2.0)	2 (1.3)	4 (2.8)	0.351
Businessman (woman) (%)	64 (21.4)	29 (18.6)	35 (24.5)	0.215
Civil servant (%)	51 (17.1)	26 (16.7)	25 (17.5)	0.851
**Ever smoked, yes (%)**	70 (23.4)	37 (23.7)	32 (22.4)	0.783
**Currently smoking, yes (%)**	18 (25.7)	11 (29.0)	7 (21.9)	0.500
**Duration of diabetes in years, mean (SD)**	5.8 (±5.9)	7.5 (±6.9)	3.9 (±3.9)	<0.001
**BMI (kg/m^2^), mean (SD)**	27.4 (±5.6)	27.5 (±5.9)	27.3 (±5.2)	0.685
**Waist circumference in cm, mean (SD)**	98.2 (±13.6)	99.9 (±14.3)	96.4 (±12.7)	0.025
**History of vigorous physical activity (≥600 METS/Week) (%)**	173 (57.9)	89 (57.1)	84 (58.7)	0.087
**Fasting blood sugar in mmol/L, mean (SD)**	11.2 (±4.8)	11.8 (±5.2)	10.5 (±4.3)	0.021
**HbA1c (%), mean(SD)**	9.7 (±6.7)	9.7 (±2.5)	9.6 (± 2.7)	0.506
**Resting heart rate in beats/min, mean (SD)**	76.6 (±13.1)	79.2 (±14.7)	73.6 (±10.4)	<0.001
**Systolic blood pressure in mmHg, mean (SD)**	141.7 (±22.6)	148.6 (±23.2)	134.2 (±19.4)	<0.001
**Diastolic blood pressure in mmHg, mean (SD)**	87.1 (±10.7)	88.8 (±10.7)	85.4 (±10.5)	0.006
**Pulse pressure in mmHg, mean (SD)**	54.6 (±17.8)	59.9 (±19.4)	48.8 (±13.8)	<0.001
**Mean arterial pressure (mmHg)**	105.3 (±13.3)	108.7 (±13.1)	101.6 (±12.5)	<0.001
**Retinopathy**				<0.001
None (%)	231 (77.3)	106 (68.0)	125 (87.4)	
Non-proliferative (%)	53 (17.7)	35 (22.4)	18 (12.6)	
Proliferative (%)	15 (5.0)	15 (9.6)	0 (0.0)	
**History of neuropathy symptoms in past 6 months**				
Palpitations, yes (%)	149 (49.8)	93 (59.6)	56 (39.2)	<0.001
Fainting or blackouts, yes (%)	114 (38.1)	63 (40.4)	51 (35.7)	0.401
‘Dead feeling’, pricking or burning sensation in the feet, yes (%)	183 (61.2)	105 (67.3)	78 (54.6)	0.024
**QTc interval (ms), mean (SD)**	428.4 (±22.3)	438.4 (±22.0)	417.5 (±16.8)	<0.001

SD: *Standard deviation*; CAN: *Cardiovascular Autonomic Neuropathy*; MET: *Metabolic Equivalent Task*.

### Prevalence and correlates of CAN

**Figure 2:**
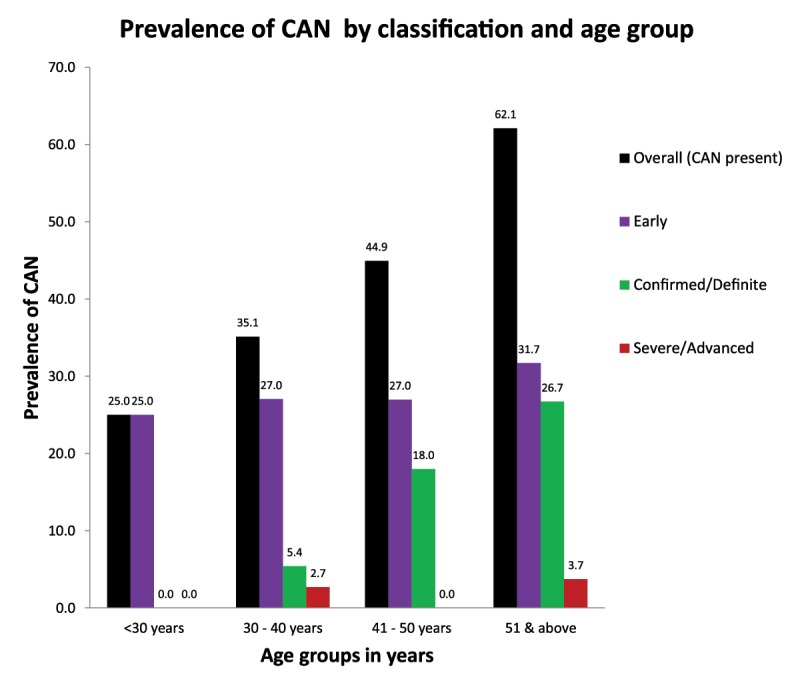
Graph showing variation of prevalence of CAN by severity with age.

CAN was diagnosed in 156/299 participants for a prevalence of 52.2% (95% CI 46.3–58.0%) on the basis of one or more abnormal cardiovascular autonomic reflex tests. The classification of CAN on the basis of CAN score is presented in Table 4. Out of 299 participants, 88 (29.4%) were classified as early CAN while 61/299 (20.4%) and 7/299 (2.3%) were classified as definite and severe (advanced) CAN respectively on the basis of the CAN score. The prevalence of CAN as assessed by the various measures of autonomic neuropathy is presented in Table 3. The most common positive sub-scale of CAN was abnormal heart rate variability with deep breathing, which we detected in 125 individuals (42%). The prevalence and severity of CAN was highest in older age groups as shown in Figure 2.

**Table 3:** Prevalence of CAN by the different assessment methods.

Test/Method	Number of cases (n)	percentage, n/N (%)
		N = 299

Resting tachycardia (pulse rate ≥100 bpm)	17	5.7
Abnormal postural index (≤1.0)	40	13.4
HRV: Reduced E/I ratio with deep breathing (<15 bpm),	125	41.8
Valsalva ratio: (<1.2), **(N** = 284)*	52	18.3
Postural hypotension	52	17.4
Diastolic BP response to sustained hand grip (<15 mmHg)	108	36.1

HRV: *Heart rate variability*; E/I: *Expiration to inspiration ratio*. * Sample size is less by 15 participants who had proliferative retinopathy and were excluded from the valsalva maneuver.

**Table 4:** Prevalence of CAN by severity (Classification of CAN).

CAN category	Number of cases (n)	Percentage, n/N (%)
		N = 299

Absent	143	47.8
Early	88	29.4
Definite/Confirmed	61	20.4
Severe/Advanced	7	2.3
**TOTAL**	**299**	**100.0**

Participants with CAN had a significantly longer duration of diabetes (p < 0.001), higher waist circumference (p = 0.025), higher systolic blood pressure (p < 0.001), higher diastolic blood pressure (p = 0.001) and higher resting pulse rate (p < 0.001) compared to those without CAN. The proportion of patients with history of palpitations (p < 0.001) and numbness in feet (p = 0.024) was significantly more among participants with CAN compared to others. The mean pulse pressure, mean arterial pressure and QTc interval were significantly higher in participants with CAN compared to those without CAN (p < 0.001).

In multivariable regression models, significant correlates of CAN were: age over 50 years (aOR 3.48, 95%CI 1.35–8.99, p = 0.010), duration of diabetes over 10 years (aOR 4.09, 95%CI 1.78-9.38, p = 0.001) and presence of diabetic retinopathy (aOR 2.25, 95%CI 1.16–4.34, p = 0.016), as shown in Table 5.

**Table 5:** Demographic and clinical factors correlated with CAN.

	% CAN positive	Univariable Analysis		Multivariable Analysis	p value

Characteristic	n/N (%)	OR 95%CI	p value	aOR 95%CI	

**Age category(years)**					
<35	7/29 (24.1)	Ref		Ref	
35–50	39/86 (45.4)	2.61 (1.01–6.75)	0.048	2.10 (0.77-5.74)	0.147
51–65	110/184 (59.8)	4.67 (1.90–11.49)	0.001	3.48 (1.35–8.99)	0.010
**Sex**					
Male	34/91 (37.4)	Ref		Ref	
Female	122/208 (58.7)	2.38 (1.43–3.95)	0.001	1.85 (0.94-3.66)	0.076
**Duration of diabetes in years**					
<5years	74/170 (43.5)	Ref		Ref	
5–9 years	38/76 (50.0)	1.30 (0.75–2.23)	0.347	1.04 (0.58–1.88)	0.893
10 years and above	44/53 (83.0)	6.34 (2.91–13.81)	<0.001	4.09 (1.78–9.38)	0.001
**Fasting blood sugar category**					
≤7.0 mmol/L	24/60 (40.0)	Ref		Ref	
>7.0 mmol/L	132/239 (55.2)	1.85 (1.04–3.29)	0.036	1.62 (0.86–3.04)	0.135
**Blood pressure category**					
≤130/80 mmHg	18/60 (30.0)	Ref			
>130/80 mmHg	105/168 (62.5)	3.89 (2.06–7.33)	<0.001		
**Waist circumference**					
Normal fat distribution	26/62 (41.9)	Ref		Ref	
Moderate central fat distribution	19/48 (39.6)	0.91 (0.42–1.95)	0.804	0.66 (0.28–1.55)	0.339
High central fat accumulation	111/189 (58.7)	1.97 (1.10–3.53)	0.022	1.16 (0.53–2.52)	0.709
**Retinopathy**					
Absent	106/231 (45.9)	Ref		Ref	
Present	50/68 (73.5)	3.28 (1.80–5.95)	<0.001	2.25 (1.16–4.34)	0.016
**History of Palpitations in past 6 months**					
No	63/150 (42.0)	Ref			
Yes	93/149 (62.4)	2.29 (1.44–3.65)	<0.001		
**Symptoms of distal peripheral neuropathy**					
No	51/116 (44.0)	Ref			
Yes	105/183 (57.4)	1.72 (1.07–2.74)	0.024		
**Resting heart rate**					
≤85 beats/min	102/227(44.9)	Ref			
>85 beats/min	54/72 (75.0)	3.68 (2.03–6.66)	<0.001		
**QTc interval**					
≤440 ms	74/213 (34.7)	Ref			
>440 ms	82/86 (95.4)	38.51 (13.58–109.21)	<0.001		

aOR: Adjusted Odds Ratio; Ref: Reference Category; CI: Confidence interval; QTc: Heart rate corrected QT.

## Discussion

In the study of ambulatory diabetic patients in Uganda, we identified a prevalence of CAN of 52.2% (95% CI 46.3–58.0%), on the basis of at least one or more abnormal cardiovascular autonomic reflex tests. In adjusted regression models, CAN was more common in those over 50, people with diabetes for over 10 years and those with diabetic retinopathy. Our results, which as far as we know are the first from the region, demonstrate a remarkably high rate of autonomic neuropathy in the East African region, and motivate an important need to better explore this preventable and morbid condition in this population.

The prevalence of CAN (52.2%) in our study is comparable with findings from several studies elsewhere in lower and middle-income countries (LMICs) that have reported values between 45% and 60% [35,36,37,38,39]. Much lower prevalence estimates of CAN were reported elsewhere, particularly in higher-income countries. For instance, prevalence estimates between 15–34% have more recently been reported in Germany, Canada, United States and England [40,41,42,43]. We suspect that the high prevalence of CAN reported in our study and others in LMICs is likely attributable to later diagnosis and poorer glycemic control in these study populations. The mean HbA1c for the study participants in the present study was 9.7% which was above the recommended target of <7.5% [44]. The variation in the prevalence also might be attributed to the different methodologies employed in the diagnosis and classification of CAN. In this study, we used cardiovascular autonomic reflex tests which are extensively used in research, and have become a reference standard for measurement of autonomic function [45], with high reported validity [46].

Both the duration of diabetes and diabetic complications such as retinopathy have also been reported as major correlates for CAN in several prior studies [7,13,15,47,48,49,50,51,52,53]. This is believed to be due to the central role of chronic hyperglycemia in the pathogenesis of CAN and other micro-vascular complications. Although the mechanisms by which hyperglycemia predisposes to CAN are not completely understood [54], increased oxidative stress, accumulation of advanced glycosylated end products, accumulation of sorbitol, disruption of hexosamine and protein C kinase pathways are the metabolic factors implicated [46,55,56]. In contrast, we did not note a correlation between glycemic control and the presence of CAN in our study. We believe this is due to the lack of a sufficient proportion of participants with adequate glycemic control in this study. However, this could also be due to reverse causality where patients become more proactive in controlling their blood sugars after developing CAN and other diabetes-related complications. This can only be detected through longitudinal studies. Moreover, because of variability in glucose control, a single point estimate of HbA1c may not correlate with the degree of autonomic neuropathy [53]. However, longitudinal studies have shown that mean HbA1c correlates with future risk of diabetic autonomic neuropathy [50,57]. Therefore, our study builds on the increasing body of evidence suggesting that other means of determining the severity of diabetes, such as the Homeostasis Model Assessment (HOMA), may be superior predictors of autonomic neuropathy [36,58].

Finally, older age is also a recognized risk factor for CAN [37,49,59]. Several changes can occur in autonomic nervous system with advanced age [60]. These changes include: selective activation of the sympathetic nervous system [61] with concomitant increase in basal plasma noradrenaline concentrations [62] and altered adrenoceptor function with reduced sensitivity to adrenergic antagonists and agonists [63]. Thus, it is well established that the autonomic nervous system function deteriorates with aging [64]. Our study hence underscores the need for targeted screening of elderly diabetic patients for CAN, so as to modulate diabetes therapeutic targets aimed at delaying the development and/or progression of CAN. The correlation with QTc interval prolongation demonstrated in our study, substantiates the recognized increased risk of adverse cardiovascular events such as cardiac arrhythmias and sudden death in patients with diabetic CAN [48,65,66].

Overall, our findings have important clinical and public health implications. We observed that most participants with diabetic CAN (88/156: 56%) had early CAN. There is emerging epidemiological evidence suggesting that there is a possibility of reversal of cardiovascular denervation in the early stages of the disease [9,67]. Therefore, improving glycemic control, life style modifications and cardiovascular disease risk factors management could slow the progression of CAN or even reverse it in this population. The high burden of CAN detected in this study also confirms the importance for early and systematic screening for this complication among patients with diabetes.

### Study Limitations

This study has some notable limitations. The study was conducted in one hospital and findings from the study may not be generalizable beyond the population of individuals in outpatient diabetes care in peri-urban Uganda. Additionally, the study was done in a regional referral hospital, and thus a referral bias may overestimate the prevalence seen in the community-based setting. That said, we excluded individuals with multi-morbidity, including known cardiovascular disease, and those >65 years old, to investigate the epidemiology of CAN in otherwise healthy individuals with diabetes. Many of our measures, including clinical history, alcohol and smoking use are self-reported, and susceptible to recall bias and social desirability bias. Another important limitation is the cross-sectional nature of the study, limiting our ability to assign the time directionality of association between CAN risk correlates (e.g. retinopathy) and CAN itself. Finally, we also are unable to assess the clinical impact of CAN without longitudinal observation of those with and without CAN, which we hope to explore in future studies. Finally, our estimates of relationships between CAN and correlates are susceptible to residual confounding of additional factors which we might not have measured or included in our regression models.

## Conclusion

We detected CAN in over half of individuals with diabetes attending an outpatient diabetes clinic in Uganda. Older age, longer duration of diabetes, and co-existence of retinopathy were key correlates of the condition. Generally, our data highlight the need for better recognition of CAN as a common complication in diabetes. We therefore recommend that screening for CAN should be considered as part of routine evaluation of diabetic individuals in ambulatory care given the high prevalence of CAN in this study population and the rising burden of diabetes. This study also emphasizes the need for better management of diabetes including improved glycemic control and regular screening for other micro-vascular complications of diabetes such as retinopathy in diabetic individuals with CAN.

## Data Accessibility Statements

The datasets generated and analyzed during the study are available from the corresponding author on request.

## Abbreviations

aOR: Adjusted Odds Ratio, BMI: Body mass index, BP: Blood pressure, CAN: Cardiovascular autonomic neuropathy, CI: Confidence interval, ECG: Electrocardiogram, HbA1c: Glycosylated hemoglobin, HOMA: Homeostatic model assessment, HRV: Heart rate variability, IQR: Inter-quartile range, MET: Metabolic equivalent task, MRRH: Mbarara Regional Referral Hospital, MUST: Mbarara University of Science and Technology, NPDR: Non-proliferative diabetic retinopathy, PDR: Proliferative diabetic retinopathy, PI: Postural Index, QTc: Heart rate corrected QT interval, SD: Standard deviation, TIDM**:** Type 1 diabetes mellitus, T2DM: Type 2 diabetes mellitus.
